# A Noninvasive Multianalytical Approach for Lung Cancer Diagnosis of Patients with Pulmonary Nodules

**DOI:** 10.1002/advs.202100104

**Published:** 2021-05-07

**Authors:** Quan‐Xing Liu, Dong Zhou, Tian‐Cheng Han, Xiao Lu, Bing Hou, Man‐Yuan Li, Gui‐Xue Yang, Qing‐Yuan Li, Zhi‐Hua Pei, Yuan‐Yuan Hong, Ya‐Xi Zhang, Wei‐Zhi Chen, Hong Zheng, Ji He, Ji‐Gang Dai

**Affiliations:** ^1^ Department of Thoracic Surgery, Xinqiao Hospital Third Military Medical University (Army Medical University) Xinqiao Main Street Chongqing 400037 China; ^2^ GeneCast Biotechnology Co., Ltd 88 Danshan Road, Xidong Chuangrong Building, Suite C‐1310 Wuxi Jiangsu 214104 China

**Keywords:** cfDNA methylation, cfDNA mutations, circulating tumor DNA, lung cancer diagnosis, machine learning, protein cancer biomarkers, pulmonary nodules

## Abstract

Addressing the high false‐positive rate of conventional low‐dose computed tomography (LDCT) for lung cancer diagnosis, the efficacy of incorporating blood‐based noninvasive testing for assisting practicing clinician's decision making in diagnosis of pulmonary nodules (PNs) is investigated. In this prospective observative study, next generation sequencing‐ (NGS‐) based cell‐free DNA (cfDNA) mutation profiling, NGS‐based cfDNA methylation profiling, and blood‐based protein cancer biomarker testing are performed for patients with PNs, who are diagnosed as high‐risk patients through LDCT and subsequently undergo surgical resections, with tissue sections pathologically examined and classified. Using pathological classification as the gold standard, statistical and machine learning methods are used to select molecular markers associated with tissue's malignant classification based on a 98‐patient discovery cohort (28 benign and 70 malignant), and to construct an integrative multianalytical model for tissue malignancy prediction. Predictive models based on individual testing platforms have shown varying levels of performance, while their final integrative model produces an area under the receiver operating characteristic curve (AUC) of 0.85. The model's performance is further confirmed on a 29‐patient independent validation cohort (14 benign and 15 malignant, with power > 0.90), reproducing AUC of 0.86, which translates to an overall sensitivity of 80% and specificity of 85.7%.

## Graphical abstract

An integrative multianalytical machine learning model based on patient clinical features, cfDNA mutation, cfDNA methylation, and protein cancer biomarkers for noninvasive cancer diagnosis of lung pulmonary nodules has achieved an area under the receiver operating characteristic curve of 0.85 on a 98‐patient discovery cohort, and 0.86 on a 29‐patient independent validation cohort, translating to 80% sensitivity and 85.7% specificity.

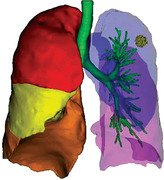

## 1 Introduction

Low‐dose computed tomography (LDCT) is commonly used to screen for lung cancer (LC) in high‐risk patients. When the original definition for a positive screen from the United States–based National Lung Screening Trial is used, LDCT performance suffered from a substantial number of positive calls (27%), of which 96% have been determined to be false positives.^[^ ^1^ ^]^ A subsequent NELSON trial then used a substantially different definition of a positive screening result to reduce the proportion of false positives to 60%, but it led to a 10‐fold decrease of sensitivity for LC detection with 2.7% positive rate.^[^ ^2^ ^]^ Distinguishing small malignant nodules in computed tomography (CT) scan from benign ones is particularly challenging because of their ambiguous radiographic characteristics.^[^ ^3^ ^]^ This issue is particularly outstanding in the authors’ hospital as it is further complicated by the relatively high prevalence of tuberculomas patients in the Chinese population.

Facing a similar dilemma, many biomarkers clinically used to broadly assess cancer risk lack the needed specificity. For example, serum‐based protein cancer biomarkers such as cancer antigen 125 (CA 125), carcinoem‐bryonic antigen (CEA), prostate‐specific antigen (PSA), and cancer antigen 19‐9 (CA 19‐9) are commonly used for monitoring lung cancer patients. But these proteins are also found in the serum of individuals without cancer which limits their clinical utility for early stage lung cancer diagnosis.^[^ ^4^ ^]^ Thus, there is an ongoing quest for biomarkers with higher specificity to supplement existing clinical practices.

Recent advance of circulating tumor DNA (ctDNA, which is one of the primary oncology research focuses based on cell‐free DNA (cfDNA)) studies offers a promising platform for noninvasive investigation of genomic alterations that are specific to the tumor. A ctDNA study typically only involves drawing liquid from the patients, such as blood, pleural fluid, cerebrospinal fluid, etc., which, compared to conventional surgery‐based biopsy approaches, incurs minimal impacts to solid tumor microenvironment, and hence avoids potential stress‐induced tumor cell proliferation. The application of next‐generation sequencing (NGS), together with advanced computational methods, has greatly enabled ctDNA‐based tumor mutational profiling in a broad range of cancer types.^[^ ^5^, ^6^ ^]^ These approaches have been successfully applied to positively diagnosed cancer patients, sometimes with reference tumor tissue sequencing profiling in order to guide treatment.^[^ ^6^, ^7^ ^]^ Meanwhile, the application of blood‐based mutational profiling for cancer screening and early detection is in its infancy, despite a number of high‐profile research publications.^[^ ^8^, ^9^, ^10^ ^]^ This is probably because the allele frequencies of these mutation targets are so low when the tumors are small^[^ ^11^, ^12^ ^]^ that they pose a challenge for reliable detection by present technologies. On lung cancer in particular, Phallen et al. compared the cfDNA mutation profiles in early‐stage lung cancer against health person, and reported they may be potential noninvasive detection biomarkers.^[^ ^13^ ^]^ However, studies on mutational biomarkers for segregating malignant lung nodules from benign ones are scarcely reported.

Global hypomethylation of DNA sequences and focal hypermethylation at CpG islands (CGIs) have been widely observed at early stages of tumorigenesis, making DNA methylation profiling an appealing approach for cancer early detection. Several studies have been reported for blood‐based screening and diagnosis of lung cancer.^[^ ^14^, ^15^, ^16^ ^]^ Ooki et al. designed a cfDNA methylation panel for early lung cancer detection, yet still based on healthy controls.^[^ ^17^ ^]^ Hulbert et al. reported that the methylation profile of the promoter regions of 6 genes can produce high diagnostic accuracy for early stage lung cancer.^[^ ^18^ ^]^ This technique was also reported with high sensitivity and specificity for the detection of early stage NSCLC in Chinese patients with small nodules.^[^ ^19^ ^]^ A clinical research reported by Liang et al. has shown that nine specific methylation markers were effective in differentiating lung cancers from benign pulmonary nodules (PNs).^[^ ^20^ ^]^ Such proof‐of‐concept work would generally require further improvement before its application to clinical practice as the performance of the test is in par with the more conventional and convenient LDCT.

A conspicuous issue with profiling using a single technology platform, being either imaging, protein biomarker, DNA mutation, or DNA methylation, is associated with the selection of biomarkers for construction of the predictive models. Given the complexity of tumor biology, a single testing platform could easily introduce systematically investigational bias to the predictive model as the observed data reflect only one aspect of the patient/sample. Moreover, clinical studies often face real‐world challenges in limited cohort size, coupling with the massive search space for potential predictive markers, the outcome models have unsurprisingly demonstrated great level of variance across different platforms and studies. Naturally, integrative analysis of multiomics data could provide a more comprehensive view of patients, reduce system bias and variance, and hence facilitate more accurate clinical decision making. A number of studies, despite few, have already shown that combining multiomics features could improve the performance of cancer screening. For example, the CancerSeek panel by Cohen et al. based on DNA point mutations and protein cancer markers has achieved a sensitivity of 59% and specificity of 99% in differentiating resectable lung cancer from normal sample.^[^ ^8^ ^]^ And the PANOPTIC classifier by Silverstri et al. based on protein cancer biomarkers and patient's clinical characteristics was able to distinguish benign from malignant lung nodules with sensitivity of 97% and specificity of 44%.^[^ ^21^ ^]^

Our study aims to advance above‐mentioned state of the art in two aspects. First, we focus on the challenging clinical application which is differentiating malignant lesions from benign ones, rather than tumor tissues against normal samples. Lesions, being pathologically altered tissues, may share a great level of molecular characteristics in comparison to normal samples, regardless of their malignancy, and hence warrant more subtle and more difficult differentiation between malignant and benign ones. Second, we evaluate the level of performance improvement through integrating higher variety of multiomics platforms including clinical feature, protein biomarker, cfDNA mutation, and cfDNA methylation to reduce systematic bias and variance, and hence improve the diagnostical efficacy of lung PNs. In this proof‐of‐concept stage, our goal was not to replace the widely adopted LDCT and its subsequent clinician's review process, but rather, to provide additional assessment metrics to assist in clinician's decision making. Given LDCT's limitation on false‐positive rate, we aimed to improve the specificity of our test for this purpose, so that, when factoring our test result together with LDCT imaging, a clinician would become more confident in excluding negative cases from subsequently unnecessary treatment.

## 2 Results and Discussion

Our study followed a typical “supervised learning” paradigm consisting of two sequential stages. In the discovery stage, various clinical and molecular features of samples on the discovery cohort were evaluated using statistical and machine learning methods to identify potential markers that are of predictive significance for PN malignancy. Then predictive models are constructed and tested with their parameters optimized. In the validation stage, the optimized predictive models are further benchmarked on an independent validation cohort to evaluate the models’ power of generalization on unknown samples. Readers shall note that due to space limitation, and with clinical practicing personnel as our targeted audience, the rest of our manuscript is slightly leaning toward the analytical results, their interpretation, and their real‐world implications in a clinical setup, rather than the algorithmic details of the computational methodology for data processing and data analysis.

### 2.1 Study Participants and Sample Characteristics

This study included tissue and plasma samples from patients screened positive for pulmonary nodules <3cm in diameter by CT scan and subsequently underwent surgical resections during February 17th, 2019—December 10th, 2019. All enrolled patients were required to be free of previous cancer history. Blood sample of sufficient quantity for this study was drawn prior to surgery, hence required a prospective observative clinical setup, although data analysis was conducted retrospectively without affecting the initial clinical decision. During discovery phase, patients were enrolled and tested without selection, so as to guarantee a real‐world positive (malignant) rate in the cohort. During validation phase, high‐risk patients (based on clinical factors) of low LDCT detection confidence (i.e., more likely negative/benign) were encouraged to enroll, in order to meet the cohort size criteria based on power analysis. Pathological assessment of PN samples was determined based on surgically resected tissue sections according to 2015 WHO Histological Classification of Lung Cancer. The collection of all samples was approved by the Ethical Committee of the Second Affiliated Hospital of Army Military Medical University (Project ID: 2019‐009‐01), and all participants provided written informed consent.

**Figure 1:**
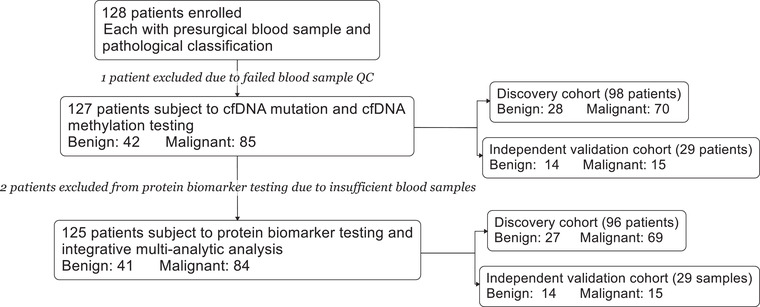
Overview of the patient cohort used in our study.

99 plasma samples were initially collected and enrolled in discovery cohort. Of them, one sample was excluded due to sample quality control (QC) failure, leaving 98 samples including 28 for patients with benign PNs and 70 malignant subject to multianalytical testing. Two patients (one benign and one malignant) did not perform protein biomarker testing and hence were excluded from protein biomarker discovery and integrative multianalytic model studies. After discovery study, a separate set of 29 samples (14 benign and 15 malignant) were enrolled in independent validation cohort (**Figure** **1**). Near the closure of the analysis, retrospectively enrolled samples also included 57 tissue samples for evaluation of the concordance of DNA methylation profiles, based on the selected methylation feature sites, between paired tissue and plasma of the same individual. Similarly, 55 tissue samples were sequenced for DNA mutations for evaluation of concordance with their paired plasma samples.

### 2.2 Clinical Features’ Correlation with PN Malignancy and Their Predictive Power

Collected clinical information of the patients included patient age, gender, smoking history, alcohol intake history, family history of cancer, nodule length, nodule width, and nodule attenuation on CT. Two nodule size calculations in forms of nodule length x nodule width, and (nodule length + nodule width)/2 were further derived. **Table** **1** summarizes the distribution of patient cohorts and their clinical features.

**Table 1:** Summary of patient demographics and clinical profiles in our study

		Discovery cohort [*N* = 98]		Independent validation cohort [*N* = 29]	
		Benign [*n* = 28]	Malignant [*n* = 70]	Benign [*n* = 14]	Malignant [*n* = 15]
Patient age		51.8±8.0	58.7±8.1	52.5±7.8	59.1±9.0
Gender	Male	16 (57.1%)	39 (55.7%)	7 (50%)	3 (20%)
	Female	12 (42.9%)	31 (44.3%)	7 (50%)	12 (80%)
Smoking history	Smokers	11 (39.3%)	30 (42.9%)	3 (21.4%)	2 (13.3%)
	Nonsmokers	17 (60.7%)	40 (57.1%)	11 (78.6%)	13 (86.7%)
Drinking history	Drinking	2 (7.1%)	13 (18.6%)	2 (14.3%)	1 (6.7%)
	Nondrinking	26 (92.9%)	57 (81.4%)	12 (85.7%)	14 (93.3%)
Family cancer history	Yes	3 (10.7%)	17 (24.3%)	3 (21.4%)	1 (6.7%)
	No	25 (89.3%)	53 (75.7%)	11 (78.6%)	14 (93.3%)
Nodule type	Solid	24 (85.7%)	52 (74.3%)	14 (100%)	14 (93.3%)
	Part‐solid	4 (14.3%)	18 (25.7%)	0 (0%)	1 (6.7%)
Nodule length [cm]		1.71±0.62	2.06±0.58	1.94±0.63	2.05±0.60
Nodule width [cm]		1.34±0.53	1.67±0.53	1.45±0.60	1.63±0.60
Pathology assessment	Carcinoid		1 (1.4%)		0 (0%)
	Squamous cell carcinoma		6 (8.6%)		0 (0%)
	Large cell carcinoma		2 (2.9%)		0 (0%)
	Small cell lung cancer		3 (4.3%)		0 (0%)
	Adenocarcinoma		58 (82.9%)		15 (100%)
	Hamartoma	5 (17.9%)		1 (7.1%)	
	Atypical adenomatous hyperplasia of the lung	1 (3.6%)		0 (0%)	
	Tuberculosis	18 (64.3%)		8 (57.1%)	
	Granulomatous inflammation	1 (3.6%)		3 (21.4%)	
	Epithelioid vascular mesothelioma	1 (3.6%)		0 (0%)	
	Sclerosing alveolar cell tumor	2 (7.1%)		1 (7.1%)	
	Castleman disease	0 (0%)		1 (7.1%)	
Stage	Ia		19 (27.1%)		6 (40%)
	Ib		41 (58.6%)		9 (60%)
	IIb		4 (5.7%)		0 (0%)
	IIIa		4 (5.7%)		0 (0%)
	IIIb		2 (2.9%)		0 (0%)

**Figure 2:**
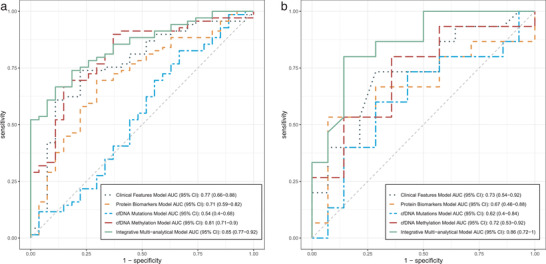
Performance of various predictive models in forms of receiver operating characteristic (ROC) curves and area under curve (AUC) scores. The performance of various predictive models based on different feature sets, namely clinical features, protein biomarkers, cfDNA mutations, cfDNA methylation, and integrated multianalytical model, respectively. The ROC curves of models are shown as lines of different colors. AUC and the 95% CI of each model are shown in the legend. a) Models’ performance on the 98‐patient discovery cohort. b) Models’ performance on the 29‐patient independent validation cohort.

On the discovery cohort, the 10 clinical features (Table S1a, Supporting Information) were assessed on their statistical significance in differentiating benign and malignant groups (Table S2, Supporting Information). Patient age consistently showed statistical significance based on our selection criteria (detailed below in the Experimental Section), with univariate prediction AUC = 0.75 on the discovery cohort (Table S2, Supporting Information) and mean AUC = 0.77 through bootstrapping (**Figure** **2**a). The positive correlation between patient age and PN malignancy is quite understandable, given that cancer risk increases as a patient get older. Additionally, one would assume that in patients of older age, their PNs would have developed longer, carrying a higher risk of malignancy.

The four nodule size measurements showed sufficient statistical significance on the discovery cohort, with AUCs slightly lower than that of patient age, and varying from 0.66 to 0.72. However, when we tested various multivariate modules using different combination of patient age and nodule size features for prediction of PN malignancy, all models performed worse than the univariance patient age model alone. This indicated that while nodule size might surface as a differentiating marker between malignant and benign nodules at the cohort level, it alone lacks the predictive power at individual sample level. This may suggest that instead of the nodule size, the cell composition and molecular characteristics of the nodule have more to do with its malignancy, and hence warrant comprehensive molecular studies in addition to CT imaging analysis.

### 2.3 Integrative Analysis of Multiple Protein Biomarkers for PN Malignancy Assessment

Eight protein cancer biomarkers most commonly used and most conveniently accessible for clinical cancer screening, namely cancer antigen 125 (CA 125), cancer antigen 15‐3 (CA 15‐3), carcinoembryonic antigen (CEA), cytokeratin‐19 fragment (CYFRA 21‐1), neuron‐specific enolase (NSE), pro‐gastrin‐releasing peptide precursor (PROGRP), squamous cell carcinoma antigen (SCC), and serum ferritin (SF) were measured Xingqiao Hospital. Based on the chemiluminescent immunoassay (CLIA) platform and following manufacturer's standard operational procedure (SOP), reagent kits being used included CA 125 II Reagent Kit (Abbott GmbH & Co. KG) for CA 125, CA 15‐3 Reagent Kit (Abbott GmbH & Co. KG) for CA 15‐3, CEA Reagent Kit (Abbott Ireland Diagnostics Division) for CEA, ARCHITECT CYFRA 21‐1 Reagent Kit (Abbott GmbH & Co. KG) for CYFRA 21‐1, ARCHITECT ProGRP Reagent Kit (Abbott GmbH & Co. KG) for PROGRP, ARCHITECT SCC Reagent Kit(Abbott GmbH & Co. KG) for SCC, Ferritin Reagent Kit (Abbott Ireland Diagnostics Division) for SF. And finally, NSE was measured by an electrochemiluminescence assay kit (ECLIA, Roche Diagnostics GmbH) following SOP.

Based on univariate analysis on the discovery cohort (Table S3a, Supporting Information), CEA, CYFRA 21‐1, and SCC have shown statistical significance (Table S4, Supporting Information), with predictive AUCs of 0.72, 0.68, and 0.67, respectively. Using these three markers, a multivariate predictive model based on support vector machine (SVM)^[^ ^22^, ^23^ ^]^ was constructed and tested on the discovery cohort with bootstrapping AUC = 0.71 (Figure 2a).

While the multivariate model had a slightly lower AUC than that of the univariance CEA, our previous empirical experiments suggested this was an acceptable and negligible performance fluctuation due to subsampling during bootstrapping, which sometimes was actually an indication that the model may have reached a relatively robust local optimization. Despite this, protein cancer biomarkers, when used alone or in combination for prediction, consistently underperformed the earlier selected clinical feature (patient age) in the same discovery cohort. While it appears somewhat surprising, it is still understandable given the well‐known lack of specificity of protein cancer biomarker testing.

### 2.4 Limitation of cfDNA Mutation Profiling for PN Malignancy Analysis

cfDNA mutation testing was conducted at GeneCast Biotechnology Co. Ltd. using a unique molecular identifier‐ (UMI‐) based and capture‐based 29‐gene NGS panel, with somatic mutations called through an in‐house bioinformatics pipeline (Experimental Section). The 29‐gene panel was designed based on curation of genes mostly related to cancer origination and development, in association with their mutation prevalence based on public databases including The Cancer Genome Atlas (TCGA) and Catalogue of Somatic Mutations in Cancer (COSMIC). After necessary QC checking and filtering based on established and validated criteria, the number of detected somatic mutations in each cfDNA sample on the discovery cohort ranged from 2 to 47, with median of 13 and average of 14.3 (Table S5, Supporting Information).

Further review of individual mutations suggested their scarce prevalence on the discovery cohort. This is probably due to the complexity of cancer origination and evolution, together with a significant percentage of noncancerous samples on the cohort. This has posted great challenge to our marker selection work, as no mutation had shown statistical significance through univariate analysis on the discovery cohort.

To tackle this obstruction, we categorized each sample's mutations into four different functional levels, based on their match to publicly available hotspots and their functional annotations (Experimental Section), and based on an assumption that mutations in the same functional level could be approximated equally in the process of cancer cell evolution. We then represented each category with two numeric features, namely the count of mutations and the maximal variant allele frequency of the mutations, respectively. This process in combination aggregated each sample's mutation profile into eight numerical features (Table S5, Supporting Information). Modeling based on these features, SVM performed AUC = 0.54 on the discovery cohort (Figure 2a).

To understand the cause to the significantly low efficacy of cfDNA mutation profiling, we further evaluated the concordance of the mutations between the paired tumor‐plasma samples, based on the additional 55 tumor sequencing cohort (26 benign and 29 malignant). Among the 60 mutations detected across the 29 malignant tumor samples, only 1 showed concordance with a total of 268 mutations detected by the correspondingly paired plasma sample, whereas among the 31 mutations for the 26 benign tumor samples, only 2 were concordant with a total of 288 in plasma. This suggested that in our specific application, the mutations detected in plasma have a poor specificity in relevance to the lesion of our particular interest, highlighting the caveats of cfDNA mutation profiling technology, not only due to the technology's limited sensitivity, but also due to the complexity of cancerous cell formation and evolution.

### 2.5 Prediction of PN Malignancy Using cfDNA Methylation Markers

**Figure 3:**
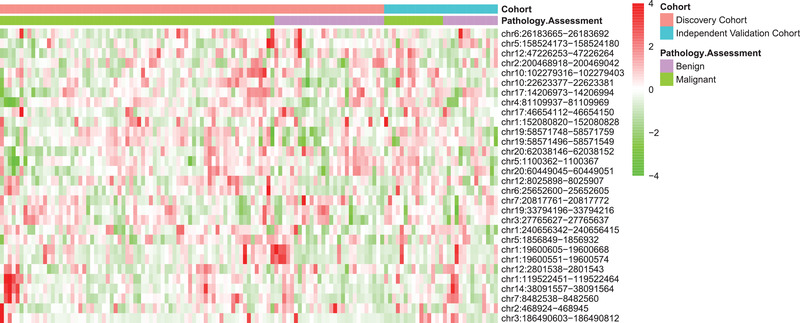
cfDNA methylation profiles of all patients in the discovery cohort and the independent validation cohort, represented using the 30‐MCB feature set. Colored bars indicate the average methylation level for the corresponding MCB.

cfDNA methylation profiling was conducted at GeneCast Biotechnology Co. Ltd using capture‐based NGS panel designed primarily based on the publicly available Cancer Genome Atlas (TCGA) dataset. Using data on the discovery set (Table S6, Supporting Information), methylated CpG sites were first clustered into 697 methylation‐correlated blocks (MCBs) for feature representation (Experimental Section). 43 MCBs showed statistical significance on the discovery cohort (Table S7, Supporting Information), of which 30 were selected as multivariate predictors through machine learning (**Figure** **3**). SVM model using the 30 MCBs performed AUC = 0.81 on the discovery cohort (Figure 2a). This is by far the best performed molecular profiling platform being investigated in our studies, consistently showing a superior sensitivity compared to protein cancer biomarkers and DNA mutations when maintained in the same level of specificity using different cut‐off values (Figure 2a).

### 2.6 Integration of Multianalytical Tests for PN Malignancy Prediction

A side‐by‐side comparison of all four models’ predictions on the discovery cohort indicated majority of consistency but a certain disagreement at the same time on each sample. Borrowing the concept of “majority voting,” we integrated all models’ predictive output through a subsequentially weighted average approach (Experimental Section). Bernoulli Naive Bayesian (BNB)^[^ ^24^ ^]^ learning model was trained on the discovery cohort, using each individual model's predictive output as its input, and the sample's pathological classification as the desired result. The integrative multianalytic BNB model has since achieved a significantly improved performance of AUC = 0.85 on the discovery cohort (Figure 2a).

It is interesting to observe that the Bayesian‐based model has outperformed any of its componential models. While patient clinical information (patient age) and protein cancer biomarker platform have shown unsatisfactory specificity, and DNA mutation platform has a very poor sensitivity, through mathematical integration, they still have contributed to achieving further performance gain on top of the DNA methylation platform, echoing an old proverb that “two hands are better than one,” and highlighting the importance of multiomics molecular testing for highly complex clinical challenges, such as PN malignancy assessment. The improved performance of the integrative model however may not appear surprising to machine learning methodology researchers. In essence, the BNB model in our study was utilized as a stacking ensemble classifier.^[^ ^25^ ^]^ Previous studies have proven that provided the first layer of input classifiers have limited correlation and are complementary to each other, which is in our case, a second layer ensemble classifier would then be able to average out noise from diverse models and thereby enhance the generalizable signal, yielding an improved accuracy.^[^ ^25^ ^]^ Further investigation of the integrative model's intermediate weights data showed that the model assigned significantly lower importance weights to protein biomarkers (mean = 0.68) and cfDNA mutations (0.71) than to cfDNA methylation (0.74) and clinical features (0.76) (Figure S3, Supporting Information), which in general agreed with each individual model's performance in our study and previous reports.^[^ ^26^ ^]^

### 2.7 Independent Validation of PN Malignancy Markers and Predictive Models

With the statistical markers identified from each testing platform, and multivariate and multianalytical models established using samples on the discovery cohort, we further benchmarked them on the 29‐patient independent validation cohort (14 benign and 15 malignant). Based on AUC of 0.85 on the discovery cohort for the final integrative model, and assuming an *α* of 0.05, a power analysis on the statistical test of the significance for AUC requires a minimal of 11 benign and 11 malignant samples to achieve adequate power of 0.9.^[^ ^27^ ^]^

The univariate clinical feature, patient age, in general maintained its predictive power on the validation cohort (Table S1b, Supporting Information) with AUC = 0.73 (Figure 2b), which translated to a sensitivity of 73.3% and specificity of 64.3% using a cut‐off age = 54 (Figure S1, Supporting Information). The age cut‐off was determined on the discovery cohort to optimize the predictive model's performance (measured as sensitivity + specificity).

On the protein cancer biomarker platform, the three selected markers (CEA, CYFRA 21‐1, and SCC, respectively) demonstrated certain univariate predictive power degradation on the independent validation cohort (Table S3b, Supporting Information), with AUCs dropping from 0.72, 0.68, and 0.67, respectively, on the discovery cohort to 0.54, 0.52, and 0.66, respectively, on the validation cohort. Their combined multivariate prediction however has held more steadily, from AUC = 0.71 on the discovery cohort to AUC = 0.67 on the validation cohort (Figure 2b), corresponding to sensitivity of 60% and specificity of 71.4% using an optimized SVM prediction score cutoff of 0.670, and reflecting a slight improvement on performance reproducibility over individual protein biomarkers. It is worthwhile to mention that our observation that the algorithmic combination of multiple protein biomarkers outperforms each individual biomarker is consistent with previous research, such as the Xpressys 13‐protein classifier for management of lung nodules that has consistent diagnostic information of ≈10% (Youden's index).^[^ ^28^ ^]^

The cfDNA mutation model that suffered from a low AUC = 0.54 on the discovery cohort maintained a same level AUC = 0.62 on the independent validation cohort (Figure 2b; and Table S5b, Supporting Information), which made it difficult to balance prediction sensitivity and specificity, yielding sensitivity = 80% and specificity = 42.9% using an optimized cut‐off threshold of 0.660 for the prediction output score.

The cfDNA methylation model has shown a certain performance degradation on the validation cohort as well, dropping from AUC = 0.81 on the discovery cohort to AUC = 0.72 on the validation cohort (Figure 2b; and Table S6b, Supporting Information), which translated to sensitivity = 93.3% and specificity = 42.9% using an optimized prediction score cut‐off of 0.606. This however is still considered a satisfactory performance in a clinical setting.

To further cross‐validate the cfDNA‐based MCB features, independent tissue DNA (tDNA) methylation sequencing were conducted on 57 patients across both discovery and validation cohorts who consented to additional tissue sequencing (27 benign and 30 malignant; and Table S8, Supporting Information), with each MCB's statistical significance assessed on this cohort based on Wilcoxon test (Figure S2a, Supporting Information). On a majority of MCBs, tDNA profiles showed a higher difference across benign and malignant groups than cfDNA, indicating these MCBs were picking malignant tumor‐specific features. At the same time, cfDNA profiles showed a high Pearson correlation coefficient of 0.84 with those of tDNA (Figure S2b, Supporting Information), strongly supporting that cfDNA‐based MCB measurements were indeed primarily derived from their paired tissue samples.

**Figure 4:**
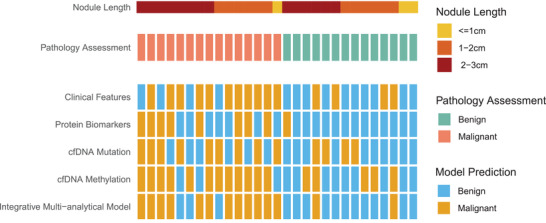
Predictive outcome of various models in comparison to the gold standard pathology assessment on the independent validation cohort. Samples were sorted according to nodule length.

Finally, in spite of varying level of performance fluctuation of each individual testing platform's predictive model, the integrative multianalytical model has steadily held its performance with AUC = 0.86 on the validation cohort (in comparison to AUC = 0.85 on the discovery cohort) (Figure 2b), corresponding to sensitivity = 80% and specificity = 85.7% using prediction score cut‐off of 0.761, striking a satisfactory balance of sensitivity and specificity, and in general significantly outperforming any individual testing platform. To further understand how the integrative model's prediction stacked with each individual model, we compared their prediction output on each validation (in terms of benign/malignant using the earlier‐mentioned optimized cut‐off thresholds) against the gold‐standard pathology assessment (**Figure** **4**). It is evidential that on the majority (19 out of 24) of samples on which the four individual models made conflicting predictions, the integrative model was able to achieve a more comprehensive assembly of each individual model's output into a correct prediction that matched the pathology assessment, proving the earlier mentioned analogy that “two hands are better than one”.

### 2.8 Correlation of the Integrative Multianalytical Model's Performance with Nodule Size

Since it is widely observed that the quantity of circulating tumor DNA in blood is positively correlated with the stage of the cancer and the tumor volume,^[^ ^29^ ^]^ we looked into the average extracted cfDNA quantity (normalized to ng mL^−1^ of whole blood) of the malignant samples and the integrative model's performance according to three different nodule length ranges, namely < = 1, >1 < = 2, and >2 < = 3, respectively (Table S9, Supporting Information).

Our data did in general supported the correlation between cfDNA quantity and nodule size, with average extracted cfDNA quantity on the discovery cohort changing from 551 ng mL^−1^ for < = 1 cm, to 613.35 ng mL^−1^ for 1–2 cm, and to 625.71 ng mL^−1^ for 2–3 cm, respectively; and on the independent validation cohort 858, 703.33, and 1015.75 ng mL^−1^, respectively. However, the integrative model's performance, measured in either AUC, sensitivity, or specificity, despite showing a certain level of fluctuation, did not support such a positive correlation (Table S9, Supporting Information). While arguably this could be due to the relatively small cohort size in our study that failed to reveal a clear statistical trend, we suspect this was also in supportive to our earlier observation that nodule size (and hence cfDNA quantity, too) was not a strong clinical predictor to PN malignancy, further strengthen our belief that molecular testing could provide a more comprehensive understanding to PN's cell composition and its malignancy than imaging per se. Additionally, our integrative multianalytical molecular testing approach has reached a sensitivity level that is generally not impacted by the small nodule size. This is evidential in Figure 4 as well, where the integrative model's performance did not show statistical bias across samples of different nodule sizes.

### 2.9 Comparison between Integrative Multianalytical Model and PET/CT in Distinguishing Malignant Nodules from Tuberculomas

**Figure 5:**
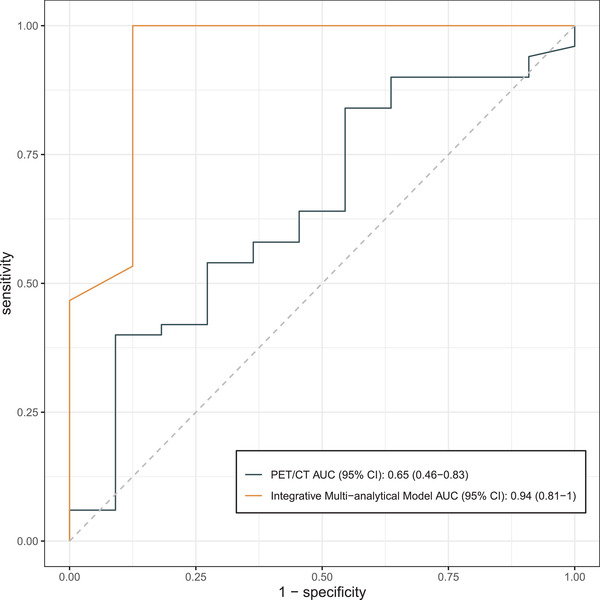
Performance comparison between PET/CT and our integrative multianalytical model, in forms of receiver operating characteristic (ROC) curves and area under curve (AUC) scores.

Molecular/anatomic imaging with ^18^F‐fluorodeoxyglucose positron emission tomography/computed tomography (PET/CT) has been widely recognized for detecting, identifying, and staging lung cancer. It provides metabolic readings, with a maximum standardized uptake value (SUVmax) >2.5 typically adopted as a cutoff value for distinguishing lung malignancies from benign diseases.^[^ ^30^, ^31^ ^]^ As a baseline reference, we studied an independent cohort of 61 patients who have taken PET/CT, of whom, 50 were later pathologically confirmed with malignant nodules and 11 with tuberculomas (Table S10, Supporting Information). While SUVmax in malignant nodules were in general higher than those in tuberculomas (7.18 ± 3.82 vs 5.36 ± 3.78), there was no statistical significance (*p* = 0.264, *t* = 1.147). Notably, should SUVmax solely be used for decision making, 9 out of 11 tuberculomas samples with SUVmax > 2.5 would have been misdiagnosed as malignant, corresponding to AUC = 0.65, sensitivity = 90%, and specificity = 9.1% (**Figure** **5**). In contrast, for the 23 patients in our independent validation cohort who had either malignant nodules (15) or tuberculomas (8), our integrative multianalytical model had AUC = 0.94, sensitivity = 80%, and specificity = 87.5% (Figure 5; and Table S11, Supporting Information). Although these two sets of performance metrics were based on two different cohorts due to the limitation that no patient in our cohort took PET/CT, and the relatively small cohorts does not speak statistical significance, they still provided some promising baseline understanding on our approach's advantage over PET/CT.

### 2.10 Cost and Availability Analysis of the Proposed Approach

While our manuscript has primarily focused on the clinical validity of the proposed approach, its real‐world economic implications shall not be overlooked before we evaluate its clinical utility. Since the primary goal of our study is to reduce over‐treatment (unnecessary surgery) of patients carrying benign lung nodules, and blood‐drawing in our approach is significantly more appealing to patients than an invasive lobectomy or sublobar resection procedure, should the total monetary cost of our combined molecular tests be lower than that of a surgery, our multiomics approach would become at least financially beneficial to the particular benign patient (in addition to all other healthcare‐related benefits). And should the total testing cost of the whole tested patient population be lower than the surgical cost of all benign patients, our approach would benefit the overall clinical field. In our study, based on the material and labor costs of our testing and an estimated industrial profit margin, the hypothetical patient‐billed testing cost is still only ≈44% of the total perioperative expense of a surgery (4000 USD vs 9000 USD). Still, this is based on the relatively large NGS panels for marker discovery in our initial experimental design. Once our small selection of predictive DNA methylation MCB markers and protein cancer biomarkers have been clinically validated in future clinical trials, it is hopeful to further cut down the testing costs. Thus, our approach has a great potential of being not only clinically feasible, but also monetarily affordable.

The second aspect of cost to patients is time—and closely related, test availability—while they await testing outcome and a go/no‐go decision on surgery. For now, the complexity of NGS‐related molecular bench work almost certainly warrants the necessity of a central molecular lab (instead of a more accessible and more rapid local point‐of‐care device), which typically requires at least several business days of turn‐around‐time (TAT). Fortunately, recent years of technological and logistical advances in NGS and molecular testing have shown that this level of testing TAT would not become a significant bottleneck in the clinical decision‐making process, as the real‐world lead time for surgical operation would typically extend beyond this TAT.

## 3 Conclusion and Future Work

LDCT's high false‐positive rate remains a challenge for diagnosis of lung PNs, which is further compounded by the relatively high prevalence of tuberculomas patients in the authors’ clinical setting. Our study adopted clinical features, protein cancer biomarkers, cfDNA mutations, and cfDNA methylation profile to garner a comprehensive profiling of PNs, and established an integrative multianalytical model in detecting malignant lung nodules from the CT diagnosis lung nodules. The model demonstrated high discriminatory power to differentiate patients with lung cancer from benign nodules on the 98‐patient discovery cohort (AUC = 0.85), which was further independently validated with AUC = 0.86 on a 29‐patient validation cohort. The integrative model significantly outperformed models based on any individual testing platform. Additionally, the model has shown significantly better performance (AUC = 0.94) in diagnosis between tuberculoma and malignant lung nodules which was a difficult task for PET/CT (AUC = 0.65). In conclusion, our study lays an effective novel approach for noninvasive diagnosis of malignant pulmonary nodules and has shown a great potential for real‐world clinical utility.

### 3.1 Added Value of Our Study

Different sets of cfDNA methylation markers have been reported in the literature for lung cancer diagnosis. Examples include the 9 cfDNA methylation sites by Liang et al.^[^ ^18^ ^]^ and the three‐gene combination model by Chen et al.^[^ ^17^ ^]^ The latter however was reported with drastically declined sensitivity and specificity with the decrease of nodule size.^[^ ^17^ ^]^ Our study used gene‐independent MCB as markers. The 30‐MCBs marker set covered larger genomic regions than previous studies, which probably was the reason that our final model maintained relatively high sensitivity and specificity regardless of nodule size, as methylation of cytosine in CpG islands silences hundreds of genes that are involved in the initiation and progression of lung cancer.^[^ ^32^ ^]^ From a data perspective, since each MCB marker is a consolidation and arithmetic mean of multiple CpG islands, the practice reduces model variance due to overfitting during marker selection and hence improves the predictive model's robustness. The similar concept, when applied to cfDNA mutation analysis, has also shown its effectiveness in handling sparse, low prevalence point‐mutation data.

Our study is probably the first that combines all clinical features, protein cancer biomarkers, cfDNA mutations, and cfDNA methylation to garner a more comprehensive understanding of PNs and hence achieve a better balance of predictive sensitivity and specificity. The Bayesian‐based integrative multianalytical model has outperformed any individual model in our study and maintained a reasonably stable and balanced sensitivity and specificity across both discovery and independent validation cohorts. Our study has clearly shown the strength of a comprehensive multiomics approach for addressing challenging clinical applications.

### 3.2 Future Work

There are several limitations of our study that warrant future work. First, the patients with pure ground glass nodules were not included in this phase, which remains a future work. Second, our model was limited to patients with pulmonary nodules screened by CT, for the purpose of assisting CT diagnosis. Its performance for cancer screening of healthy people is yet to be assessed. Third, our model is for prediction on malignant nodules and invasive cancer, and its diagnostic effect on preinvasive lesions, such as minimally invasive adenocarcinoma and adenocarcinoma in situ needs further verification. And finally, despite having been independently validated on a 29‐patient cohort, the predictive performance of our model, and hence the efficacy of our approach remains to be further validated in a much larger and more diverse patient population, a work that is already underway and expected to be reported in a follow‐up manuscript.

## 4 Experimental Section

##### Sample Collection

10 mL of blood was drawn and stored in Cell‐Free DNA Storage Tube (PET) (cwbiotech Cat# CWY025M) at room temperature. Blood was centrifuged at 1600 rpm for 10 min at room temperature and separate plasma in a new tube with a pipette. Plasma was centrifuged at 12 000 rpm for 15 min at 4 °C, and separate supernatant in a new tube with a pipette. Plasma was separated from blood (no apparent hemolysis) within 72 h after blood draw and stored at −80 °C until DNA isolation.

##### cfDNA Extraction

cfDNA was extracted using the MagMAX Cell‐Free DNA Isolation (Thermo Fisher Cat#A29319) per manufacturer instructions. Germline DNA was isolated from PBMCs using the TIANamp Blood DNA Kit (TIANGEN). At least 10 ng cfDNA was required both for DNA and methylation sequencing libraries preparation. Tissue genomic DNA (gDNA) was isolated from malignant and benign FFPE lung tissue samples using the MagPure FFPE DNA LQ Kit B (Magen Cat# D6323‐02B) according to the manufacture's protocol. At least 100 ng gDNA was required for methylation sequencing library preparation.

##### cfDNA Mutation Profiling

cfDNA mutation sequencing was based on a lab‐developed capture panel test at GeneCast Biotechnology Co. Ltd., that has been internally validated following College of American Pathologists (CAP) guidelines. Briefly, cfDNA mutation sequencing libraries were prepared using KAPA HyperPrep Kit (Roche), based on adapters with unique molecular identifiers (UMIs). After adapter ligation, DNA was hybridized to an in‐house designed 29‐gene mutation panel of 124 Kb size, using xGen Hybridization and Wash kit (IDT). The final sequencing libraries were quantified using Qubit dsDNA HS Assay Kit (Thermo Fisher Cat#Q32854). DNA libraries were sequenced on an Illumina NovaSeq 6000 sequencing system paired‐end 151bp read length.

##### cfDNA Mutation Calling

Point mutations including SNVs, InDels and multinucleotide variants (MNV) were called using a validated in‐house UMI‐aware bioinformatics pipeline that incorporates a number of publicly available and internal software tools including Illumina bcl2fastq (https://support.illumina.com/sequencing/sequencing_software/bcl2fastq‐conversion‐software.html) for sequencing reads demultiplexing, Trimmomatic^[^ ^33^ ^]^ for base quality trimming, BWA‐mem^[^ ^34^ ^]^ for sequencing mapping to hg19 reference genome (http://hgdownload.cse.ucsc.edu/goldenpath/hg19/bigZips/hg19.fa.gz), fgbio tools (https://github.com/fulcrum‐genomics/fgbio) for UMI processing, pysam (https://github.com/Debian/pysam) for mapped reads processing and variant calling, etc. Sample‐matched WBCs and an internal reference variant database of normal samples were used to identify matched germline SNPs and clonal hematopoiesis mutations. An internal sequencing specific error database was also used to remove false positive variants due to laboratory processing and sequencing artifacts.

##### cfDNA Mutation Classification

cfDNA mutations were classified into four different levels. The single residue or in‐frame indel mutation hotspot identified in the 24 592 tumor samples listed in the Memorial Sloan Kettering Cancer Center Cancer Hotspots database (https://www.cancerhotspots.org) was used as a core hotspot list. Level 1 mutations were defined as oncogene variants showing in the core hotspot list. Level 2 mutations were either tumor suppressor gene variants in the core hotspot list, or any other deleterious tumor suppressor gene variants as annotated by SIFT^[^ ^35^ ^]^ and PolyPhen.^[^ ^36^ ^]^ The remaining exonic nonsynonymous mutations were classified as Level 3. And all other mutations fell in Level 4.

##### cfDNA Methylation Profiling

cfDNA Methylation sequencing was conducted at GeneCast Biotechnology Co., Ltd based on a 1.16 Mb capture panel designed surrounding over 95 000 CpG sites that were selected based on publicly available The Cancer Genome Atlas (TCGA) data. Methylation libraries were prepared using Accel‐NGS Methyl‐Seq DNA Library Kit (Swift Biosciences Cat#30 096). Hybridization to the capture panel was carried out using SeqCap EZ Hyb and Wash Kit (Roche Cat# 5 634 253 001). And the purification was accomplished using Seq Cap EZ Pure Capture Bead Kit (Cat# 6 977 952 001). The final sequencing libraries were quantified using Qubit dsDNA HS Assay Kit (Thermo Fisher Cat#Q32854). Methylation libraries were sequenced on an Illumina Xten Sequencing System with read length of paired‐end 151bp.

##### cfDNA Methylation Data Processing

Methylation sequencing reads were processed using an in‐house pipeline that contained Illumina bcl2fastq for demultiplexing, Trimmomatic for base quality trimming, BisMark^[^ ^37^ ^]^ for reference genome mapping, BisMark, Samtools, and BamUtil (https://github.com/statgen/bamUtil) for mapped reads deduplication, sorting and clipping. Reads with mapping quality less than 20 and conversion rate less than 95% were filtered out. Methylation level of CpG sites were called by BisSNP^[^ ^38^ ^]^ in the form of beta‐value. For quality control, CpG sites with depth <100x were removed and beta‐values of sites <2 supporting methylated reads were replaced by 0. A MCB is defined as a genomic region that contains at least 3 CpG sites, each site with < = 100 bp distance and > = 0.9 Pearson's correlation coefficient to its adjacent site. Correlations between each pair of neighboring CpG sites were calculated for benign and malignant groups in the discovery cohort respectively. Only those blocks with high correlations in both two groups were considered. 697 MCBs were generated under the criteria, consisting of 4678 CpG sites. The mean beta‐value of CpG sites in an MCB was used as the methylation level of the MCB.

##### Univariate Analysis for Marker Selection

For data based on clinical features, cancer protein biomarkers, and cfDNA methylation MCBs, six univariate tests including ANOVA, Fisher's exact test, Chi‐Square test, Wilcoxon rank sum test, Mann–Whitney test, and Student's *t*‐test were conducted on the discovery cohort to assess each variable's differentiating power between the benign and the malignant sample groups. *p* < 0.1 in each test was considered statistically significant. A variable is considered as a candidate marker only if it is statistically significant in at least four of the six tests. The univariate predictive AUC was also calculated for reference purpose but not used as a marker selection criterion. All univariate tests were conducted using R version 3.6.3 (https://www.r‐project.org/).

##### Machine Learning for Nodule Malignancy Classification

Each numeric data point *x* was first normalized to log2(*x *+ 1) for outlier control and Gaussian distribution approximation. Missing data point was imputed with median value of the corresponding feature readings on the discovery cohort. Data were finally standardized using z‐score, which is calculated as *z* = (*x* – mean(*X*))/std(*X*) where X is all reading of *x* on the discovery cohort. For methylation MCB features, Recursive Feature Elimination with Cross‐Validation (RFECV)^[^ ^22^ ^]^ was used to conduct additional feature selection in order to optimize the model's accuracy on the discovery cohort. This process was implemented through in‐house Python (version 3.7) scripts based on machine‐learning package *scikit‐learn*. Each round of the RFECV process works by recursively removing lower‐ranked features from the candidate feature set and evaluate through cross‐validation the performance of the remaining features, until an optimized performance is achieved. Through 20 stratified shuffled‐split cross‐validator with 10 splitting iterations and a range of 20–40% test size, 30 MCBs out of the initially screened 43 candidates were selected as the consensus feature set for subsequent training. A classifier based on SVM^[^ ^23^ ^]^ implemented in the same in‐house Python software kit was trained with its performance assessed based on 13‐fold cross‐validation. SVM works by optimizing the parameters for a predefined type of hyperplane (termed as kernel) that separates the benign and malignant classes in the study to maximize the total distance between all training data points to the hyperplane, in other words, to optimize the separation of the two classes. A straightforward liner kernel was used in the study. In each fold, hyper‐parameter optimization was tuned by a cross validation grid exhaustive search, with randomly selected 60% samples on the discovery cohort for training and the remaining 40% for testing and refitted the training cohort with the best score parameters.

##### Integrative Multianalytical Model

Each individual model's predictive output on each study field (namely clinical features, cancer protein markers, cfDNA mutations, and cfDNA methylation) consisted a probability score of being malignant. The four scores were used as the input of a subsequent BNB^[^ ^24^ ^]^ model that was trained to optimize the assignment of “voting power” (i.e., importance weight) to each individual model in order to fit each data point's known classification label. BNB is a probabilistic algorithm based on probability theory and Bayes’ Theorem to predict the probability of an unknown input belonging to predefined classes. In lay language, since the performance of each individual classifier on the training dataset (i.e., the probability of the input sample's malignance being correctly predicted when the classifier outputs a certain output score) is known, it would be able to derive a weighted average formula to calculate the probability of an input sample being malignant based on the combination of all four classifiers’ prediction scores. The algorithm was implemented in the same above‐mentioned in‐house Python toolkit.

##### Statistical Analysis

Due to the prospective observative nature of the study, no statistical analysis was performed to determine the discovery cohort size, which was largely affected by sample availability. Patient enrollment was stopped for discovery based on the performance analysis of the predictive markers and models, cofactoring the overall project timeline. Power analysis for determining the size of the independent validation cohort was done using *One ROC Curve Power Analysis* module of *PASS 2020* software, based on the model of Obuchowski and McCLISH.^[^ ^27^ ^]^

## Conflict of Interest

The authors declare no conflict of interest.

## Supporting information

Supporting Information

Supplemental Table 1

Supplemental Table 2

Supplemental Table 3

Supplemental Table 4

Supplemental Table 5

Supplemental Table 6

Supplemental Table 7

Supplemental Table 8

Supplemental Table 9

Supplemental Table 10

Supplemental Table 11

## Data Availability

The data that support the findings of this study are available in the supplementary material of this article. Raw sequencing data are openly available at https://bigd.big.ac.cn/bioproject/browse/PRJCA003983 or from the author.
