# Supplementary material for: A Noninvasive Multianalytical Approach for Lung Cancer Diagnosis of Patients with Pulmonary Nodules
Source: Adv Sci (Weinh). 2021 May 7;8(13):2100104. doi: 10.1002/advs.202100104 (PMC8261512; doi:10.1002/advs.202100104)
Supplement: Supplementary file 1 — Supporting Information [file ADVS-8-2100104-s009.pdf]

© 2021 Wiley-VCH GmbH

ADVANCED SCIENCE
Open Access

Supporting Information

for *Adv. Sci.*, DOI: 10.1002/advs.202100104

A Non-invasive Multi-analytical Approach for Lung Cancer Diagnosis of Patients with Pulmonary Nodules

Quan-Xing Liu, Dong Zhou, Tian-Cheng Han, Xiao Lu, Bing Hou, Man-Yuan Li, Gui-Xue Yang, Qing-Yuan Li, Zhi-Hua Pei, Yuan-Yuan Hong, Ya-Xi Zhang, Wei-Zhi Chen, Hong Zheng, Ji He*, Ji-Gang Dai**

A Non-invasive Multi-analytical Approach for Lung Cancer Diagnosis of Patients with Pulmonary Nodules

Quan-Xing Liu[#], Dong Zhou[#], Tian-Cheng Han[#], Xiao Lu[#], Bing Hou, Man-Yuan Li, Gui-Xue Yang, Qing-Yuan Li, Zhi-Hua Pei, Yuan-Yuan Hong, Ya-Xi Zhang, Wei-Zhi Chen, Hong Zheng[*], Ji He[*], Ji-Gang Dai[*]

[#]These authors contributed equally to this work.

Dr. Q. Liu, Dr. D. Zhou, Dr. X. Lu, Dr. B. Hou, Dr. M. Li, Dr. G. Yang, Dr. Q. Li, Dr. H. Zheng, Prof. J. Dai

Department of Thoracic Surgery, Xinqiao Hospital, Third Military Medical University (Army Medical University), Xinqiao Main Street, Chongqing 400037, China.

E-mail: ziecoe@tmmu.edu.cn, daijigang@tmmu.edu.cn

T. Han, Dr. Q. Li, Dr. Z. Pei, Dr. Y. Hong, Dr. Y. Zhang, Dr. W. Chen, Dr. J. He

GeneCast Biotechnology Co., Ltd, 88 Danshan Road, Xidong Chuangrong Building, Suite D-401, Wuxi City, Jiangsu 214104, China.

E-mail: he.ji@genecast.com.cn

Online-only Figure legends

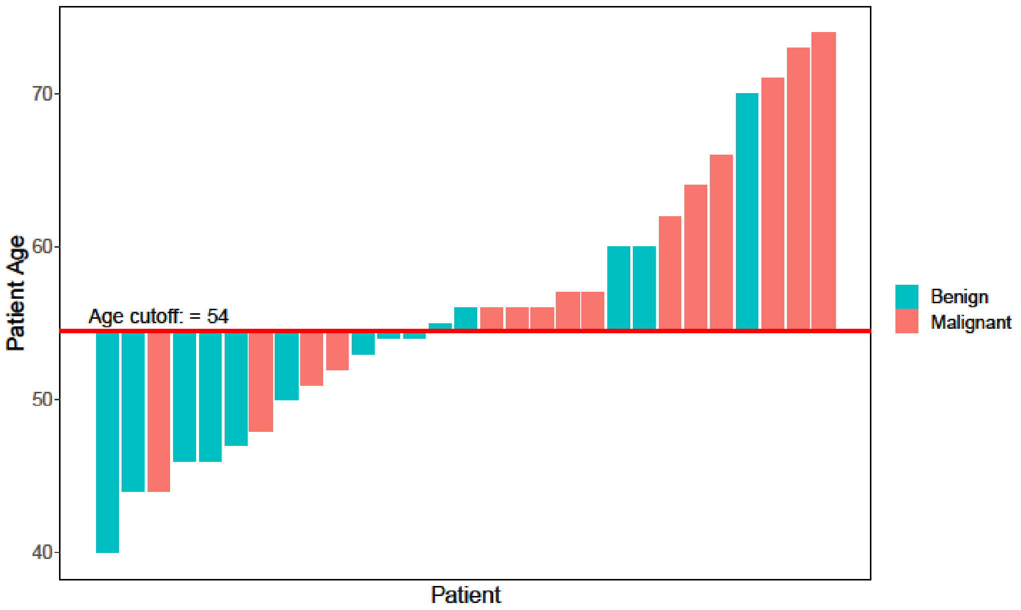

Figure S1. Correlation of patient age with PN malignancy validated on the independent validation cohort and presented in waterfall chart. An age cut-off of 54 derived from the discovery cohort had a sensitivity of 73.3% (11/15) and specificity of 64.3% (9/14) on the independent validation cohort.

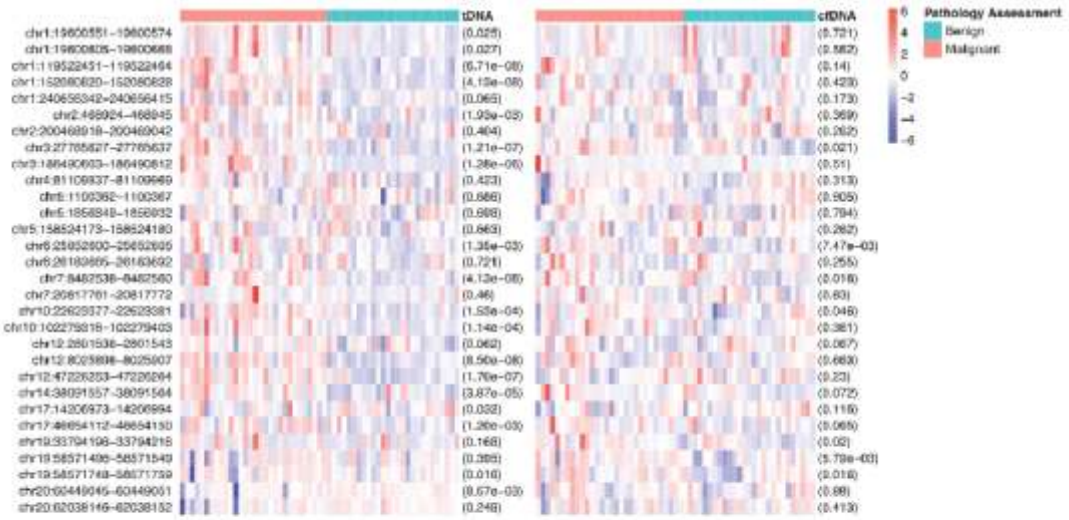

(a)

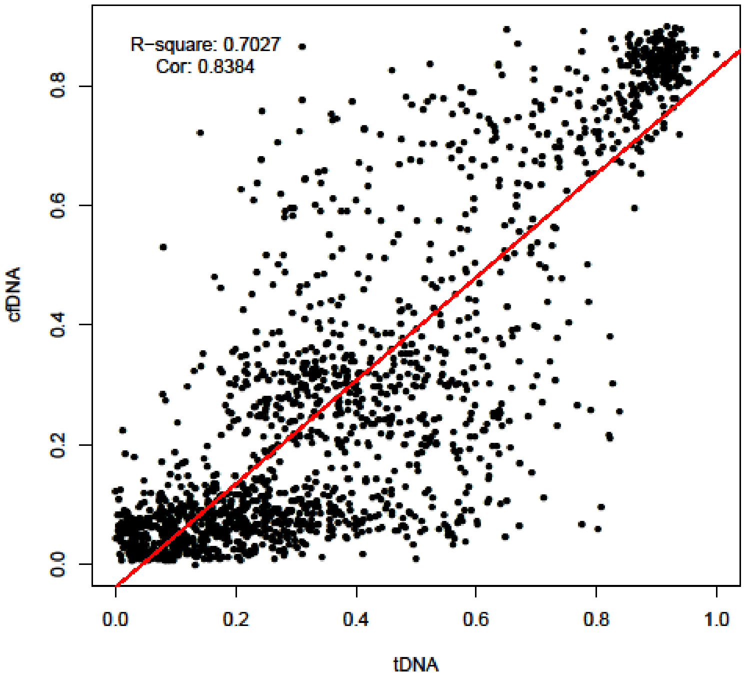

(b)

Figure S2. Correlation of cfDNA-based with tDNA-based methylation profiles. (a). Heatmap of cfDNA and tDNA methylation profile heatmaps based on the selected 30 MCB markers. (b). Pearson correlation coefficient between cfDNA-tDNA measurements. Data were based on a 57-patient cohort with paired tumor-blood samples.

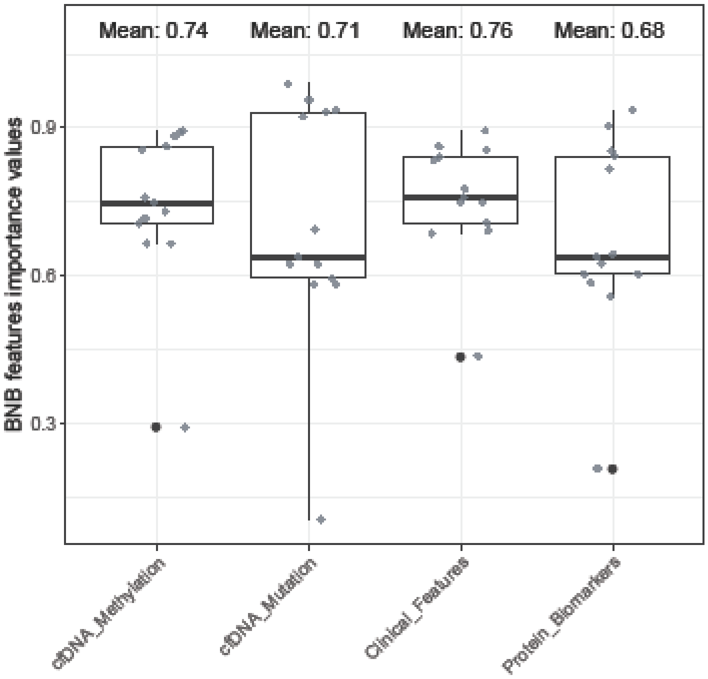

Figure S3. Importance weight of each individual model assigned by the integrative multi-analytic model.

Online-only Table legends

Table S1. Clinical features of the patients on (a) discovery cohort, and (b) independent validation cohort.

Table S2. Statistical significance of clinical features between benign and malignant samples on the discovery cohort.

Table S3. Protein biomarker profiles of the samples on (a) discovery cohort, and (b) independent validation cohort.

Table S4. Statistical significance of protein biomarkers between benign and malignant samples on the discovery cohort.

Table S5. cfDNA mutation features of the patients on (a) discovery cohort, and (b) independent validation cohort.

Table S6. cfDNA methylation profiles of samples on (a) discovery cohort, and (b) independent validation cohort. The methylation profile was represented with beta values of 697 MCBs.

Table S7. Statistical significance of 697 MCBs between benign and malignant samples on the discovery cohort.

Table S8. List of patient IDs on the 57-patient cohort with both tissue DNA (tDNA) and cell-free DNA (cfDNA) methylation sequencing.

Table S9. Average extracted cfDNA quantity of the malignant samples and the integrative multi-analytic model's performance according to different ranges of nodule lengths on (a) discovery cohort, and (b) independent validation cohort. cfDNA quantity was normalized to ng/mL of whole blood.

Table S10. PET/CT results of an independent 61-patient cohort.

Table S11. Integrative multi-analytical model's prediction results of 23 patients on the independent validation cohort.
